# Quantification of Droplet Aerosol Generation During Phacoemulsification and Pars Plana Vitrectomy

**DOI:** 10.7759/cureus.101100

**Published:** 2026-01-08

**Authors:** Basil Suresh, Harikesh Kaneshayogan, Zixia Liu, James Haywood, Daniel Partridge, Jonathan Park, Edward Herbert, Inderpaul Sian

**Affiliations:** 1 General Medicine, West Hertfordshire Teaching Hospitals NHS Trust, Watford, GBR; 2 Ophthalmology, West Hertfordshire Teaching Hospitals NHS Trust, Watford, GBR; 3 Mathematics and Statistics, University of Exeter, Exeter, GBR; 4 Ophthalmology, Taunton and Somerset NHS Foundation Trust, Somerset, GBR

**Keywords:** aerosol generating procedure, covid 19, pars plana vitrectomy, s: cataract surgery, surgical techniques

## Abstract

Introduction

Intraocular procedures such as phacoemulsification and pars plana vitrectomy (PPV) may generate fine droplet aerosols that are relevant to infection control, particularly in the context of SARS-CoV-2. Data on aerosol production during cataract and vitrectomy surgery, especially in human tissue and with different wound constructions, remain limited. This study used a high-sensitivity optical particle spectrometer to quantify droplet aerosols (0.12-8.00 μm in diameter) generated during phacoemulsification in cadaveric human eyes with 2.2 mm and 2.75 mm corneal incisions, to assess whether hydroxypropyl methylcellulose (HPMC) reduces aerosol production, and to measure aerosol generation during individual steps of PPV.

Methods

Tests were performed on one model eye and two human cadaveric eyes. A printed optical particle spectrometer (POPS) was used to measure droplet aerosol generation during phacoemulsification through 2.2 mm and 2.75 mm main corneal incisions, with and without HPMC coating, and during predefined stages of 23-gauge PPV. Particle number concentration (PNC, particles cm⁻³) was recorded each second and summarised as mean PNC for each condition.

Results

In this small series, mean PNC during phacoemulsification without HPMC appeared to be higher with 2.75 mm incisions than with 2.2 mm incisions, and counts of particles >1 μm in diameter were also greater. Application of HPMC was associated with reduced aerosol counts. The maximum measured mean PNC without HPMC for 2.2 mm corneal incisions was 88 cm⁻³, which fell to 66 cm⁻³ with HPMC (p<0.05). For 2.75 mm incisions, the maximum measured mean PNC without HPMC was 493 cm⁻³, falling to 61 cm⁻³ with HPMC (p<0.05). No increase in droplet aerosol was detected during vitrectomy apart from during air infusion through a leaking trocar valve.

Conclusion

To our knowledge, this is the first study to use whole cadaveric human eyes in combination with a high-sensitivity optical particle spectrometer to quantify airborne particle number and size during phacoemulsification and PPV. In this model, droplet aerosol production during cataract surgery appeared lower with 2.2 mm incisions and with HPMC coating of the cornea. Droplet aerosols may be generated during vitrectomy when air infusion is delivered through a leaking trocar valve, highlighting the importance of port integrity.

## Introduction

The SARS-CoV-2 pandemic has raised concern about aerosol-generating procedures in healthcare, including ophthalmic surgery. Intraocular procedures such as phacoemulsification and pars plana vitrectomy (PPV) may generate fine droplet aerosols that are relevant to infection control, particularly when respiratory viruses are present in ocular tissues or fluids [1-4]. Aerosols are airborne fine solid particles or liquid droplets, and particles <10 μm in diameter are of particular concern because they can remain suspended for longer and contribute to airborne transmission [2]. SARS-CoV-2 has been reported to persist in aerosols with a half-life of one hour; thus, reducing the production of aerosols during invasive procedures may help protect patients and staff [5]. 

Cataract extraction via phacoemulsification is the most commonly performed surgical procedure in the National Health Service (NHS), and PPV is used to treat a range of acute and chronic sight-threatening conditions. Ocular manifestations of SARS-CoV-2, as well as detection of viral RNA in ocular secretions and tissues, suggest that intraocular surgery could, in principle, pose a transmission risk to theatre staff in infected patients [3,4]. Several experimental studies using model, porcine, and sheep eyes have begun to characterise droplet and aerosol generation during phacoemulsification and vitrectomy [6-11]. However, most have relied on visualisation of larger, visible droplets or on particle counters in non-human tissue, and data using human cadaveric eyes with high-sensitivity aerosol measurement are limited. 

In this exploratory pilot study, we used human cadaveric eyes and a high-sensitivity optical particle spectrometer to quantify droplet aerosols generated during phacoemulsification with 2.2 mm and 2.75 mm corneal incisions, with and without hydroxypropyl methylcellulose (HPMC) coating, and to assess droplet aerosol production during predefined stages of 23-gauge PPV. The primary goal was to describe aerosol patterns under these experimental conditions.

## Materials and methods

Study design and specimens

Testing was performed on a model eye and subsequently on two human cadaveric eyes. Given the limited number of cadaveric eyes and the pilot nature of the study, no formal sample size calculation was performed, and the study was not powered to detect statistically significant differences. All statistical analyses were therefore considered exploratory and descriptive, with p-values reported as aids to interpretation rather than as evidence of confirmatory hypothesis testing. The model cataract eye (Philips Studio model cataract eyes, PS-012) was used in a model head, which was placed in a Perspex box with an open front and top (dimensions 100 × 50 × 40 cm). The box was used to reduce particles entering the operating field from the sides. The cadaveric specimens were whole, non-fixed human eyes, obtained from NHS Blood and Transplant as research-designated tissue. These were deemed unsuitable for clinical use according to standard eye bank criteria and were supplied without known prior intraocular surgery or active ocular infection. Corneas and sclera were intact on gross inspection.

Aerosol detection and instrumentation

A Printed Optical Particle Spectrometer (POPS; NOAA Chemical Sciences Laboratory), a high-sensitivity optical particle counter, was used to detect droplet aerosol generation during surgery [12]. POPS samples air through an inlet into a chamber, where particles are illuminated by a 405 nm laser and detected by a photomultiplier tube. Particle size is inferred from light-scattering amplitude using Mie theory. The instrument measures particles across 200 size channels spanning approximately 0.12-8.00 μm at a sample flow rate of 3 cm³/s. The POPS unit was placed inside the Perspex box at a fixed distance from the eye, and before each series of measurements for a given eye, it was run for 10 minutes in standard operating theatre conditions to characterise background aerosol. Particle number concentration (PNC, particles cm⁻³) was then recorded once per second over 60 seconds for each operative step.

Experimental setup

A 2.2 mm temporal corneal incision was made, and phacoemulsification was performed. Changes in particle detection were measured, and the optimal position for the probe was determined to be 2.5 cm from the eye. Once the optimal setup was achieved, a second model cataract eye was then used with a 2.2 mm incision to carry out the quantification experiments. Phacoemulsification was performed using the Alcon Constellation system (power 80%, torsional amplitude 15-75%, flow 23 cc/min), and droplet aerosol production was measured initially without HPMC and then with HPMC (HPMC USP 2.0%, Bio-Tech Vision Care, Pvt Ltd, India) applied over the cornea, covering the incision. The above was then repeated on a human cadaveric eye (NHS Blood and Transplant) in the same order. The incision was enlarged to 2.75 mm in the same eye, and the same steps were carried out (with and without HPMC). Once phacoemulsification was complete, three self-sealing 23-gauge trocar ports (Alcon product reference: 8065751801, Alcon Laboratories Inc., USA) were inserted into the eye at 4 mm from the limbus. Core vitrectomy was carried out with settings of 5000 cuts per minute, infusion pressure of 25 mmHg, and vacuum set at 450 mmHg.

Droplet aerosol measurements were taken at the following stages: 1) vitrectomy with vitrector inside the eye; 2) vitrectomy with vitrector exiting the fluid-filled eye whilst cutting; 3) vitrector exiting the fluid-filled eye with cutting switched off; 4) fluid infusion on with a leaking valve and no active manoeuvres; 5) during fluid-air exchange; 6) with air infusion (set at 35 mmHg infusion pressure) on with a leaking valve and no active manoeuvres. For the second cadaveric eye, the 6 steps for vitrectomy were performed first, followed by 2.2 mm incision phacoemulsification with and without HPMC covering the corneal incision, and finally 2.75 mm incision phacoemulsification with and without HPMC. This assessed whether prior intraocular surgery would result in a difference in droplet aerosol production.

Outcome measures and statistical analysis

PNC was expressed as particles per cubic centimetre (PNC cm⁻³). For each experimental condition (baseline, phacoemulsification with and without HPMC for 2.2 mm and 2.75 mm incisions, and vitrectomy stages S1-S6), we calculated the mean PNC and standard deviation, as well as the maximum (“peak”) PNC observed during the 60-second trace. A secondary descriptive analysis was performed for larger particles by summing channels corresponding to particles >1 μm in diameter. To explore whether aerosol generation differed between surgical conditions, we compared the distributions of one-second PNC values between baseline and each experimental condition using two-sided paired t-tests within each eye. Paired comparisons were performed for phacoemulsification with vs. without HPMC for a given incision size and for vitrectomy stages S1-S6 vs. baseline. P-values (with a nominal threshold of 0.05) are reported to indicate the strength and direction of observed differences and should be interpreted cautiously in light of the pilot design and limited sample size, without implying confirmatory statistical inference. All analyses were performed using Python, and graphs were generated using Igor Pro (WaveMetrics Inc., Oregon, USA).

Taunton and Somerset NHS Foundation Trust confirmed that ethical review was waived because only previously deemed unsuitable cadaveric tissue was used and no live participants or clinical care pathways were involved.

## Results

Phacoemulsification surgery 2.2 mm incision vs. 2.75 mm incision, with and without HPMC

**Figure 1:**
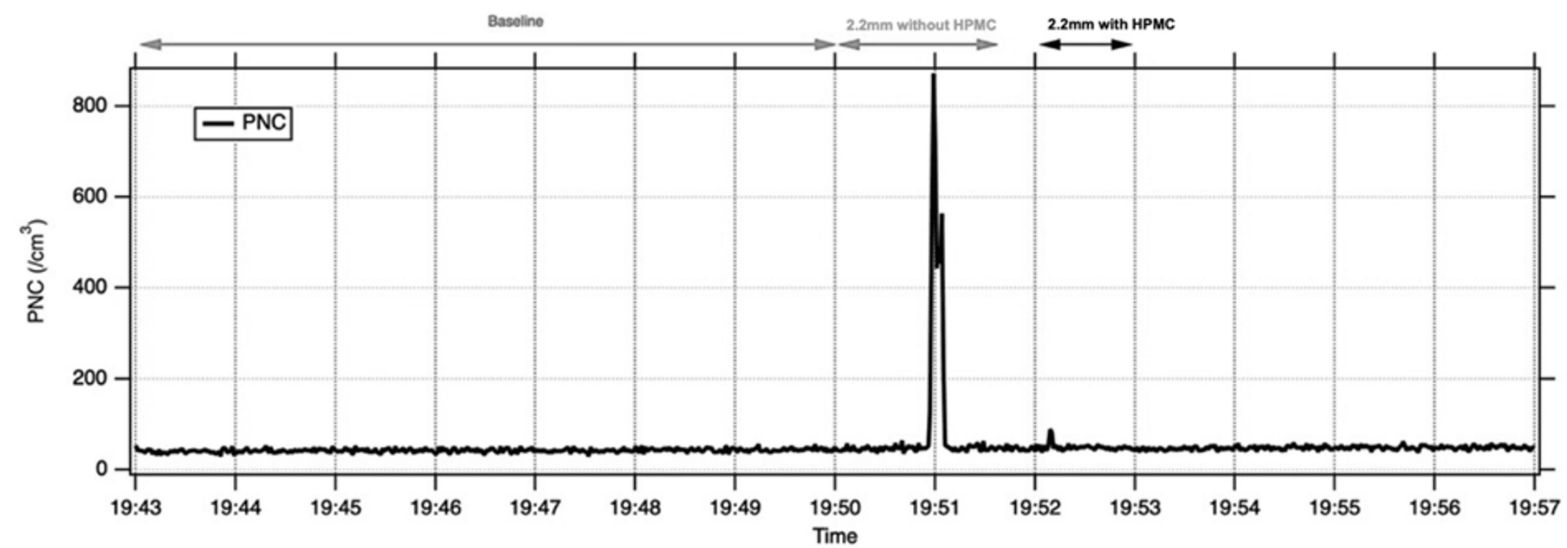
Increase in PNC during 2.2 mm incision phacoemulsification without HPMC in a model eye, which is reduced on application of HPMC

**Figure 2:**
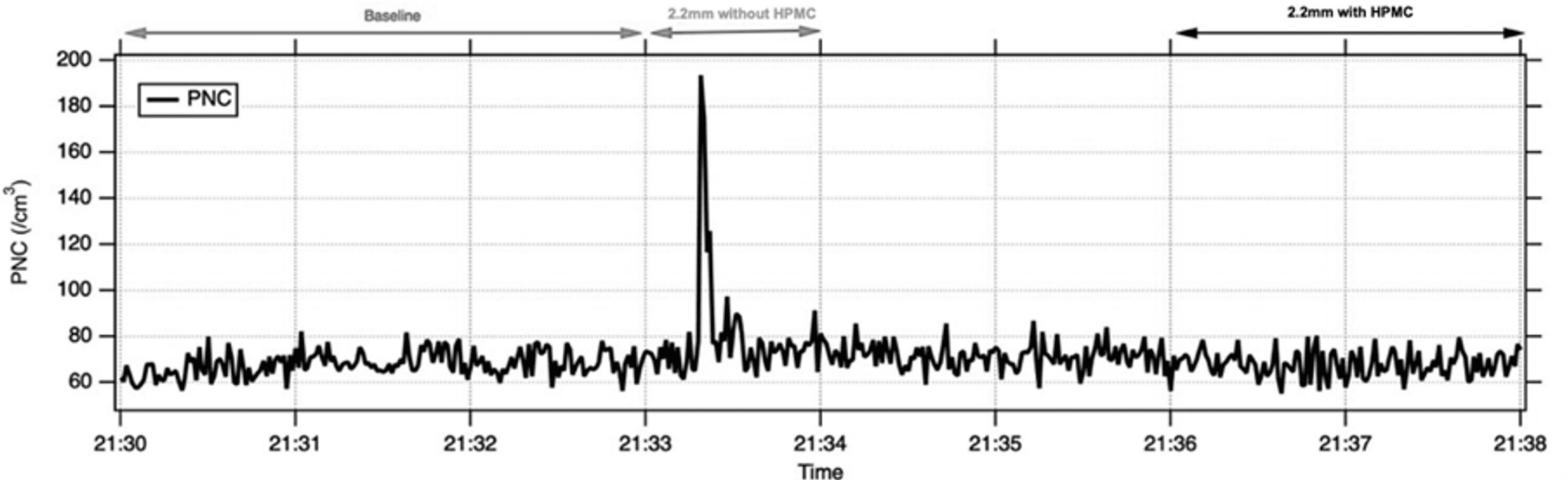
Increase in detected PNC during 2.2 mm incision phacoemulsification without HPMC in a cadaveric eye (Eye 1), which is reduced on application of HPMC

**Figure 3:**
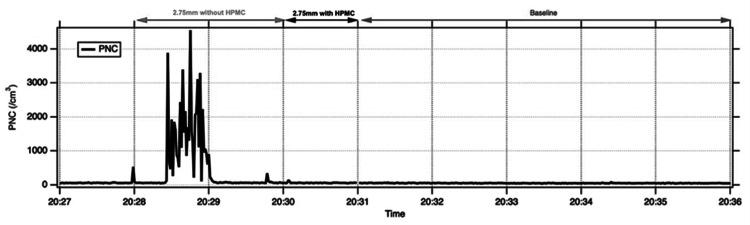
Increase in detected PNC during 2.75 mm incision phacoemulsification without HPMC in a cadaveric eye (Eye 1), which is reduced on application of HPMC

Mean PNCs for each condition are shown in Table 1 and representative time-series traces in Figures 1-3. With a 2.2 mm corneal incision, mean PNC during phacoemulsification without HPMC was higher than baseline in the model eye and both cadaveric eyes (model eye 91 ± 148 vs. 42 ± 4 cm⁻³; cadaveric eye 1: 88 ± 94 vs. 68 ± 5 cm⁻³; cadaveric eye 2: 79 ± 23 vs. 68 ± 6 cm⁻³). Application of HPMC over the cornea reduced mean PNC towards baseline in all three eyes. In both cadaveric eyes, HPMC use was associated with a statistically detectable reduction in PNC compared with phaco without HPMC (exploratory paired t-test, p<0.05). Time-series plots demonstrate transient spikes in aerosol counts during 2.2 mm phaco without HPMC, with peak PNCs up to 871 cm⁻³ in the model eye and 746 and 192 cm⁻³ in cadaveric eyes 1 and 2, which were markedly attenuated after HPMC application (Figures 1, 2).

**Table 1:** Mean particle number concentration summary Mean (± standard deviation) particle number concentration (PNC, particles cm⁻³) during phacoemulsification using 2.2 mm and 2.75 mm corneal incisions, with and without hydroxypropyl methylcellulose (HPMC), in the model eye and two cadaveric eyes.

	^2.2 mm incision^			^2.75 mm incision^		
	^Baseline (PNC cm-3)^	^Without HPMC (PNC cm-3)^	^With HPMC (PNC cm-3)^	^Baseline^ ^(PNC cm-3)^	^Without HPMC^ ^(PNC cm-3)^	^With HPMC^ ^(PNC cm-3)^
^Model Eye^	^42±4^	^91±148^	^50±8^	^n/a^	^n/a^	^n/a^
^Cadaveric Eye 1^	^68±5^	^88±94^	^66±6^	^54±8^	^493±883^	^61±12^
^Cadaveric Eye 2^	^68±6^	^79±23^	^68±6^	^53±7^	^65±7^	^62±5^

For 2.75 mm incisions, measurements were available for the two cadaveric eyes. In cadaveric eye 1, mean PNC increased from a baseline of 54 ± 8 cm⁻³ to 493 ± 883 cm⁻³ during phacoemulsification without HPMC and fell to 61 ± 12 cm⁻³ after HPMC was applied (exploratory paired t-test, p<0.05; Table 1, Figure 3). In cadaveric eye 2, mean PNC with a 2.75 mm incision remained close to baseline, with or without HPMC. These inter-eye differences should be interpreted cautiously. We did not systematically vary or quantify wound construction or tissue properties, and potential measurement artefacts (for example, intermittent condensation on the detector probe) cannot be excluded. Across eyes and incision sizes, size-resolved analysis showed that larger particles contributed disproportionately to the observed increases, with HPMC addition bringing the number of large droplet aerosols back to baseline.

Vitrectomy surgery

**Figure 4:**
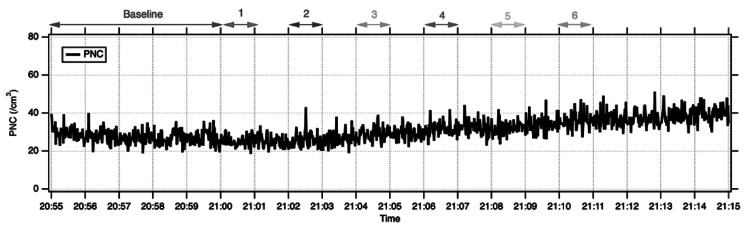
No increase in detected PNC during all stages of surgery in a cadaveric eye (eye 1)

**Figure 5:**
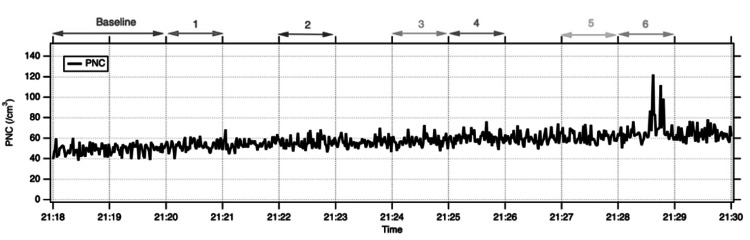
Increase in detected PNC during stage 6 (air exchange through a leaking trocar valve) in a cadaveric eye (eye 2) PNC: Particle number concentration

Mean PNCs during the stages of PPV are summarised in Table 2. In cadaveric eye 1, mean PNC remained close to baseline across all stages, and no discernible increase in aerosol counts was observed on the time-series trace. In cadaveric eye 2, overall PNC levels were slightly higher, but again, there was no clear rise above baseline during instrument entry or exit, cutting, or fluid-air exchange (Figure 4). A short-lived increase in PNC was detected during stage 6 in cadaveric eye 2, when air infusion was delivered through a deliberately leaking trocar valve. During this phase, peak PNC reached 122 cm⁻³ for particles 0.4 - 7 μm in diameter (Figure 5), although the 60-second mean PNC remained within the same order of magnitude as baseline. Overall, droplet aerosol production during PPV did not show a clear increase above baseline, apart from a spike during air infusion with a leaking valve (Figure 5).

**Table 2:** Mean particle number concentration during stages of pars plana vitrectomy Mean (± standard deviation) particle number concentration (PNC, particles cm⁻³) for baseline and vitrectomy stages S1–S6 in two cadaveric eyes. Vitrectomy stages are defined in the Methods.

	Mean PNC (cm-3)						
	Baseline	S1	S2	S3	S4	S5	S6
Cadaveric Eye 1	28±4	25±3	26±4	29±4	32±4	32±4	36±4
Cadaveric Eye 2	50±5	53±5	56±5	57±5	60±5	61±5	69±12

## Discussion

This pilot study detected droplet aerosols during specific steps of cataract and vitrectomy surgery. In one cadaveric eye, 2.75 mm corneal incisions without HPMC were associated with substantially higher PNC than 2.2 mm incisions, whereas in the second eye, PNC remained close to baseline across incision sizes. These findings, observed in a single cadaveric eye, are compatible with the hypothesis that larger incisions may permit greater aerosol escape; however, the small sample size and inter-eye variability mean that this should be regarded as exploratory. These findings suggest that, in experimental settings, HPMC application may reduce droplet aerosol generation, supporting previous work indicating that a viscoelastic coating can provide a physical barrier against the release of droplets from corneal incisions [13]. Our POPS-based cadaveric data are consistent with model-eye and quantitative particle-counter studies showing that phacoemulsification can generate aerosols under experimental conditions, while live-surgery work using optical particle counters has not demonstrated a significant increase in small-particle aerosols above baseline. These data suggest that any aerosol generated during routine phacoemulsification is modest and may not translate into high clinical risk [14,15].

We also observed a short-lived increase in aerosol counts during 23-gauge PPV when air infusion was delivered through a deliberately leaking trocar valve. This contrasts with reports by Liyanage et al. and Koshy et al., who did not observe visible droplets during vitrectomy, although both assessed macroscopic droplet formation only, and Liyanage et al. used 25-gauge instruments [6,9]. Wong et al. demonstrated visible droplets during 25-gauge vitrectomy on model eyes [7]. These prior studies relied on high-resolution video, with or without fluorescein-stained absorbent paper, and therefore predominantly detected larger, super-micron droplets with high water content. Smaller, submicron aerosolised particles may sediment more slowly and escape through incisions without being detected visually. The advantage of our approach is the use of a particle spectrometer capable of detecting particles 0.12-1 μm in diameter, which is likely to be more sensitive for capturing fine droplet aerosols overall.

Our findings also differ from those of Lee et al., who did not detect aerosol generation during phacoemulsification in porcine eyes using 2.8 mm incisions [8]. The optical particle counter used in that study had a lower detection limit of 0.3 μm and six preset size channels, whereas the POPS instrument in our work measured particles down to 0.12 μm across 200 size channels. Similar detectors to those used by Lee et al. were used by Naveed et al., who did not detect aerosol production during 23- and 25-gauge vitrectomy in porcine eyes [11], and by Okada et al., who reported no aerosol in sheep eyes [10]. Differences in tissue (human cadaveric vs. porcine or sheep), incision construction, and instrument gauge, as well as the greater sensitivity and size resolution of POPS, may all contribute to the discrepancies between our results and these animal studies. However, our small sample size means that some of the observed differences could also reflect sampling variability.

The potential infectious relevance of these aerosols depends on whether viable virus is present within intraocular tissues and fluids. The presence of SARS-CoV-2 RNA in ocular tissue has been confirmed in some reports and not detected in others [16-18]. Koo et al. detected RNA in the aqueous humour of 6/31 (19%) asymptomatic patients undergoing elective surgery, and Casagrande et al. found genomic and subgenomic RNA in the cornea and occasionally in aqueous and vitreous samples from viremic patients [19,20]. Together with retinal biopsy data showing SARS-CoV-2 RNA in the posterior segment, these studies suggest that ocular and intraocular structures can harbour viral genetic material in at least a proportion of infected individuals [21]. However, most report low RNA loads and rarely demonstrate viable virus, so the implications for transmissible infection remain uncertain.

Many centres employ pre-operative screening pathways for elective surgery, including symptom assessment, self-isolation and SARS-CoV-2 testing, and many patients undergoing emergency eye surgery are also swabbed prior to theatre where feasible. These measures are likely to reduce the proportion of infectious patients presenting for intraocular surgery. During phacoemulsification, the virucidal effect of povidone-iodine and ongoing intraocular irrigation may further lower any anterior segment viral load. Within this context, our findings suggest that choosing smaller corneal incisions (2.2 mm rather than 2.75 mm, where surgically appropriate) and applying HPMC to coat the corneal wound may provide additional, low-cost strategies to reduce droplet aerosol generation during cataract surgery.

In our experiments, the magnitude of droplet aerosol production during vitrectomy was substantially lower than during phacoemulsification, with peak PNC values during PPV of 122 cm⁻³ compared with spikes reaching several hundred to several thousand particles per cm³ during phaco. The small increase we observed during air infusion through a leaking trocar valve suggests that optimising valve integrity may help to minimise aerosol escape at this stage, although these strategies were not directly evaluated in this study and should be regarded as conceptual rather than evidence-based recommendations. Other potential strategies to reduce SARS-CoV-2 exposure in high-risk individuals include deferring surgery where clinically safe and considering extraocular approaches such as scleral buckling if appropriate. Any such decisions, however, need to balance the theoretical reduction in aerosol exposure against the risks associated with longer operating times or general anaesthesia, including closer proximity of the patient’s airway to theatre staff.

Aerosol generation during PPV in our model was confined to a very short phase of surgery and appeared to arise predominantly from the infusion line at the leaking valve, rather than from intraocular tissue manipulation itself. If intraocular viral RNA levels are low, as suggested by several studies, the total amount of infectious material contained within such brief aerosol bursts is likely to be limited [16-18]. Because the risk of infection relates to both infectious dose and duration of exposure, these observations are broadly reassuring, although they do not eliminate the possibility of transmission in high-risk scenarios [22]. Particle concentrations also decrease with distance from the source as droplets sediment out of the air, although smaller particles can travel further before settling [23]. Our measurements were obtained with the detector positioned 2.5 cm from the eye, considerably closer than the typical distance between the surgical field and the surgeon’s face. It is therefore plausible that aerosol concentrations at the level of the surgeon’s airway are lower than those recorded in this study. Considering the modest aerosol counts we observed, their short duration, and the attenuation of particle concentrations with distance, our findings are consistent with a limited contribution of intraoperatively generated droplet aerosols to SARS-CoV-2 transmission risk during intraocular surgery. However, we did not measure viral RNA in aerosolised particles, so these inferences remain speculative and should be interpreted with caution.

This study does have key limitations worth highlighting. First, only two cadaveric eyes could be sourced during the peak of the pandemic, and the number of repeated measurements per eye was limited by progressive tissue degradation, which may reduce generalisability to live surgery. No formal sample size calculation was undertaken, and the number of eyes reflected tissue availability during the pandemic rather than a priori power considerations. Second, cadaveric ocular tissue may not accurately reproduce the wound biomechanics and fluid dynamics of living eyes. Third, all measurements were made at a single detector position, so aerosol dispersion patterns at other locations, including the surgeon’s position, were not assessed. Fourth, although the POPS particle counter is well validated, each measurement is subject to experimental error, and with a small number of eyes, any sampling bias may be magnified. Finally, we measured particle number and size but did not assess viral RNA or viability, so infectious risk can only be inferred indirectly. 

## Conclusions

In this cadaveric human-eye model, phacoemulsification (particularly with 2.75 mm incisions and without HPMC) generated measurable fine droplet aerosols, whereas vitrectomy produced only low-level, transient aerosols mainly during air infusion through a leaking trocar valve. These aerosols could, in principle, carry respiratory pathogens, but their modest magnitude and short duration, together with likely low intraocular viral loads and distance-related dilution, suggest that the overall risk of SARS-CoV-2 transmission to theatre staff from intraocular surgery is probably low. Use of smaller corneal incisions where feasible, HPMC wound coating, careful attention to trocar integrity, and context-appropriate personal protective equipment may provide simple, pragmatic measures to further mitigate risk, particularly in high-risk or SARS-CoV-2-unknown cases. This pilot cadaveric study supports further investigation rather than direct clinical extrapolation. Future work should include a larger series of cadaveric and, where feasible, in vivo intraoperative measurements to better characterise inter-eye variability, different wound constructions and gauges, and to correlate aerosol profiles with direct virological assessment of intraocular samples.
